# Mortality risk stratification in antineutrophil cytoplasmic antibody-associated vasculitis: baseline severity, accumulated damage burden and cardio-renal vulnerability in a Chinese two-center cohort

**DOI:** 10.3389/fmed.2026.1909872

**Published:** 2026-07-22

**Authors:** Lina Zhang, Jing Zhang, Penghui Xu, Sheng-Guang Li, Huiqiong Zhou

**Affiliations:** 1 Department of Rheumatology and Immunology, Peking University International Hospital, Beijing, China; 2 Department of Rheumatology and Immunology, The Fourth Medical Center of PLA General Hospital, Beijing, China

**Keywords:** antineutrophil cytoplasmic antibody-associated vasculitis, Birmingham Vasculitis Activity Score, cardio-renal vulnerability, Five-Factor Score, mortality, renal involvement, risk stratification, Vasculitis Damage Index

## Abstract

**Objective:**

Mortality in antineutrophil cytoplasmic antibody-associated vasculitis (AAV) is influenced by active inflammation, organ-threatening severity, irreversible damage and host vulnerability. We evaluated how BVAS-2003, FFS-2009 and VDI relate to all-cause mortality, and compared their endpoint discrimination with a baseline cardio-renal vulnerability model informed by the DANGER framework.

**Methods:**

We retrospectively studied 122 patients with AAV from two tertiary rheumatology centers. BVAS-2003 and FFS-2009 were assessed at diagnosis. VDI was updated during follow-up and was interpreted as accumulated damage burden rather than as a time-fixed baseline predictor. Overall survival was estimated using Kaplan–Meier analysis. Endpoint associations were assessed using a parsimonious multivariable logistic model, and discrimination was compared using receiver operating characteristic curves.

**Results:**

During a median follow-up of 36 months, 36 patients died. Estimated 1-, 3- and 5-year survival rates were 78, 58 and 39%, respectively. Non-survivors had higher BVAS-2003, higher FFS-2009, higher accumulated VDI, lower hemoglobin, higher serum creatinine and more frequent dialysis. In the endpoint multivariable model, FFS-2009 and VDI retained associations with all-cause mortality, whereas BVAS-2003 did not retain an independent association after adjustment. The DANGER-variable model showed numerically highest discrimination (area under the curve 0.78), followed by VDI (0.74), FFS-2009 (0.73) and BVAS-2003 (0.71).

**Conclusion:**

In this severe real-world AAV cohort, mortality stratification was associated with baseline organ-threatening severity, endpoint accumulated damage burden and cardio-renal vulnerability more than with global activity alone. Because VDI was not measured at a uniform landmark, VDI-related findings should be interpreted as endpoint damage-burden associations rather than validated prospective prediction.

## Introduction

Antineutrophil cytoplasmic antibody-associated vasculitis (AAV) is a potentially life-threatening group of small-vessel vasculitides, including granulomatosis with polyangiitis, microscopic polyangiitis and eosinophilic granulomatosis with polyangiitis. Although modern immunosuppression has improved survival, excess mortality persists, especially among patients with renal failure, pulmonary involvement, infection, cardiovascular comorbidity and treatment-related complications (1–3). Contemporary management recommendations emphasize remission induction, infection prevention, renal protection and glucocorticoid-sparing strategies in severe disease (4–7). Practical mortality stratification remains clinically important because early recognition of high-risk patients can support closer surveillance, infection prevention, renal support planning and glucocorticoid-sparing strategies.

Existing vasculitis instruments measure different clinical constructs. BVAS-2003 quantifies current inflammatory activity across organ systems and is useful for assessing disease burden and treatment response (8). FFS-2009 was developed as a mortality-oriented severity index that emphasizes prognostically important features, including older age, renal insufficiency, cardiac involvement, severe gastrointestinal disease and absence of ear-nose-throat involvement (9). VDI records irreversible damage attributable to disease or therapy and is conceptually distinct from active inflammation (10). These instruments are often used together in clinical practice, but their prognostic meaning differs because they operate on different time axes.

Previous studies have suggested that FFS and early damage assessment may be more closely related to survival than global activity scores alone (11–13). However, the magnitude and independence of these associations vary across cohorts, and disease activity, renal function, age, infection and conventional comorbidity may all contribute to mortality risk (2, 3, 14). In addition, PR3-ANCA and MPO-ANCA serotypes define clinically meaningful AAV subsets and have been associated with differences in clinical phenotype, relapse tendency and outcome, supporting their inclusion in baseline characterization (15–17). More recently, a DANGER score for ANCA-associated renal vasculitis highlighted the prognostic importance of readily available baseline cardio-renal variables, including age, hemoglobin, renal function and cardiovascular comorbidity (18). These variables are particularly relevant to cohorts with substantial renal involvement and may capture host vulnerability not fully represented by vasculitis activity indices.

The novelty of the present study is not the derivation or validation of a new score, but a construct-sensitive and time-aware comparison of routinely available clinical instruments in a real-world Chinese AAV cohort. We aimed to: (i) compare BVAS-2003, FFS-2009 and VDI in relation to all-cause mortality; (ii) evaluate a DANGER-variable baseline cardio-renal model as a clinically intuitive comparator; and (iii) clarify how diagnosis-time severity, longitudinal accumulated damage burden and host vulnerability should be interpreted separately when counseling and monitoring patients with AAV.

## Materials and methods

### Study design and patients

This retrospective observational study included 122 consecutive patients diagnosed with AAV between January 2015 and December 2024 at two tertiary rheumatology centers in Beijing, China. Patients were eligible if they fulfilled the 2012 revised Chapel Hill Consensus Conference nomenclature and accepted classification criteria for granulomatosis with polyangiitis, microscopic polyangiitis or eosinophilic granulomatosis with polyangiitis (19–22). The two centers manage a high proportion of severe systemic and renal AAV, and the cohort should therefore be interpreted as a tertiary-care real-world cohort rather than a population-based inception cohort.

Baseline data were abstracted from electronic medical records, including age, sex, disease subtype, ANCA specificity, organ involvement, hemoglobin, serum creatinine and comorbidities. Follow-up continued until death, last clinical contact or 31 December 2024. Eosinophilic granulomatosis with polyangiitis was included to reflect routine practice; sensitivity analyses excluding these patients were performed because their biology and clinical phenotype differ from granulomatosis with polyangiitis and microscopic polyangiitis.

### Clinical scores and damage assessment

BVAS-2003 (23) and FFS-2009 (9) were assessed at diagnosis, before or at the start of induction therapy. FFS-2009 was analyzed both as a continuous score and in clinically relevant categories. BVAS-2003 was analyzed continuously and by cohort-informed cut-offs for visual stratification. The high-activity threshold of 24 points was selected because it approximated the upper part of the baseline BVAS distribution in this cohort; this threshold was used for visualization and not proposed as a universal cut-off.

VDI was assessed during follow-up and updated at the last available visit or immediately before death (10). Because standardized 6-month or 12-month damage scores were not routinely recorded and could not be reliably reconstructed, VDI was not treated as a time-fixed baseline predictor. The cut-off of VDI ≥4 approximated the upper damage distribution and was used to visualize high accumulated damage burden; tertile-based VDI plots were also provided to reduce dependence on a single cohort-derived threshold. This distinction is central to the present analysis: FFS and BVAS describe diagnosis-time severity and activity, whereas VDI describes damage accrued during the observed disease course.

### Treatment and supportive-care variables

Treatment exposure and supportive-care variables were extracted when available. These included cyclophosphamide, rituximab and mycophenolate mofetil exposure, initial glucocorticoid dose, cumulative glucocorticoid dose, glucocorticoid pulse therapy, dialysis requirement and maintenance dialysis. Missing treatment data were handled with available-case denominators and no imputation. Because treatment choices in retrospective vasculitis cohorts are strongly affected by confounding by indication, treatment variables were reported descriptively rather than used as causal predictors.

### Outcome

The primary outcome was all-cause mortality. Deaths were identified from hospital records and, when possible, confirmed through registry or administrative sources. Causes of death were recorded from death summaries and clinical notes but were not centrally adjudicated. When infection was recorded as the principal cause of death, available information on primary site, infection type and whether infection occurred during active vasculitis or remission was reviewed; however, these details were not uniformly available across centers.

### Statistical analysis

Continuous variables are presented as mean and standard deviation or median and interquartile range, as appropriate. Categorical variables are presented as numbers and percentages. Survivors and non-survivors were compared using Student’s *t* test or the Mann–Whitney U test for continuous variables and the chi-square test or Fisher’s exact test for categorical variables. All analyses were exploratory and were designed to compare clinical constructs rather than to establish causal effects of individual covariates.

Overall survival was estimated by the Kaplan–Meier method. Survival curves were compared with log-rank tests for prespecified strata defined by FFS-2009, BVAS-2003, VDI and the combined FFS-by-VDI risk grid. The survival analyses were used to visualize risk separation across clinically interpretable strata. Because VDI was assessed after baseline and was partly dependent on observation duration, VDI-based survival curves were interpreted descriptively as damage-burden strata rather than as causal evidence that baseline VDI predicted subsequent death.

A parsimonious multivariable logistic regression model was used to evaluate endpoint all-cause mortality by the end of follow-up. Although Cox regression is generally preferred for time-to-event analyses based on time-fixed baseline predictors, a standard Cox model treating VDI as a baseline covariate would have been conceptually inappropriate because VDI was measured during follow-up. Serial VDI dates and standardized early damage landmarks were not available to support a valid time-dependent Cox model. Therefore, the logistic model was retained only as an index-based endpoint association model and was not interpreted as a fully adjusted causal prediction model.

The endpoint logistic model included age at diagnosis, BVAS-2003, FFS-2009 and VDI. Model complexity was deliberately restricted because only 36 deaths occurred. Hypertension and cardiovascular history were incorporated into a separate DANGER-variable model, while diabetes mellitus was reported descriptively because adding multiple traditional risk factors to the index-based model would have increased overfitting risk. The DANGER-variable model was specified as: logit[P(death)] = beta0 + beta1(age) + beta2(hemoglobin) + beta3(serum creatinine) + beta4(history of hypertension or cardiovascular disease). This approach compared the component variables of the published DANGER framework without recalibrating or externally validating the original points-based score. Discrimination was compared with receiver operating characteristic curves and areas under the curve. Analyses were performed in SPSS version 26 and R version 4.2. Two-sided *p* values below 0.05 were considered statistically significant.

## Results

### Cohort characteristics and mortality

The cohort included 122 patients: 61 with granulomatosis with polyangiitis, 49 with microscopic polyangiitis and 12 with eosinophilic granulomatosis with polyangiitis. Mean age at diagnosis was 61.6 years and 66 patients were male. Baseline renal involvement was common. ANCA specificity distribution broadly paralleled clinical phenotype: 61 patients were PR3-ANCA-positive, 49 were MPO-ANCA-positive and 12 were ANCA-negative. The mean BVAS-2003 was 21.7, the mean FFS-2009 was 1.1 and the mean final VDI was 3.8 (Table 1).

**Table 1:** Baseline characteristics by survival status in the antineutrophil cytoplasmic antibody-associated vasculitis cohort (*N* = 122).

Characteristic	Overall (*N* = 122)	Survivors (*n* = 86)	Non-survivors (*n* = 36)	*p* value
Age at diagnosis, years	61.6 ± 15.5	60.4 ± 15.7	64.4 ± 15.0	0.130
Male sex, *n* (%)	66 (54.1)	45 (52.3)	21 (58.3)	0.500
Granulomatosis with polyangiitis, *n* (%)	61 (50.0)	46 (53.5)	15 (41.7)	0.200
Microscopic polyangiitis, *n* (%)	49 (40.2)	32 (37.2)	17 (47.2)	0.300
Eosinophilic granulomatosis with polyangiitis, *n* (%)	12 (9.8)	8 (9.3)	4 (11.1)	0.700
PR3-ANCA-positive, *n* (%)	61 (50.0)	46 (53.5)	15 (41.7)	0.200
MPO-ANCA-positive, *n* (%)	49 (40.2)	32 (37.2)	17 (47.2)	0.300
ANCA-negative, *n* (%)	12 (9.8)	8 (9.3)	4 (11.1)	0.700
BVAS-2003, score	21.7 ± 8.6	19.8 ± 7.9	26.1 ± 8.7	<0.001
FFS-2009, score	1.1 ± 0.9	0.9 ± 0.7	1.6 ± 1.0	<0.001
Final VDI, score	3.8 ± 2.4	3.2 ± 2.1	5.3 ± 2.6	<0.001
Hemoglobin, g/L	104.3 ± 24.6	108.3 ± 24.2	94.8 ± 23.2	0.005
Serum creatinine, μmol/L	247.7 ± 303.7	211.3 ± 281.3	334.6 ± 340.1	0.016
Hypertension, *n* (%)	45 (36.9)	26 (30.2)	19 (52.8)	0.019
Diabetes mellitus, *n* (%)	23 (18.9)	12 (14.0)	11 (30.6)	0.032
Coronary heart disease, *n* (%)	15 (12.3)	8 (9.3)	7 (19.4)	0.140
Current smoker, *n* (%)	45 (36.9)	28 (32.6)	17 (47.2)	0.130
Regular alcohol use, *n* (%)	35 (28.7)	24 (27.9)	11 (30.6)	0.800
At least one traditional risk factor, *n* (%)	53 (43.4)	31 (36.0)	22 (61.1)	0.011

Values are mean ± standard deviation or *n* (%). *p* values compare survivors with non-survivors. ANCA specificity distribution broadly paralleled clinical phenotype in this cohort. ANCA, antineutrophil cytoplasmic antibody; BVAS, Birmingham Vasculitis Activity Score; FFS, Five-Factor Score; MPO, myeloperoxidase; PR3, proteinase 3; VDI, Vasculitis Damage Index.

**Figure 1:**
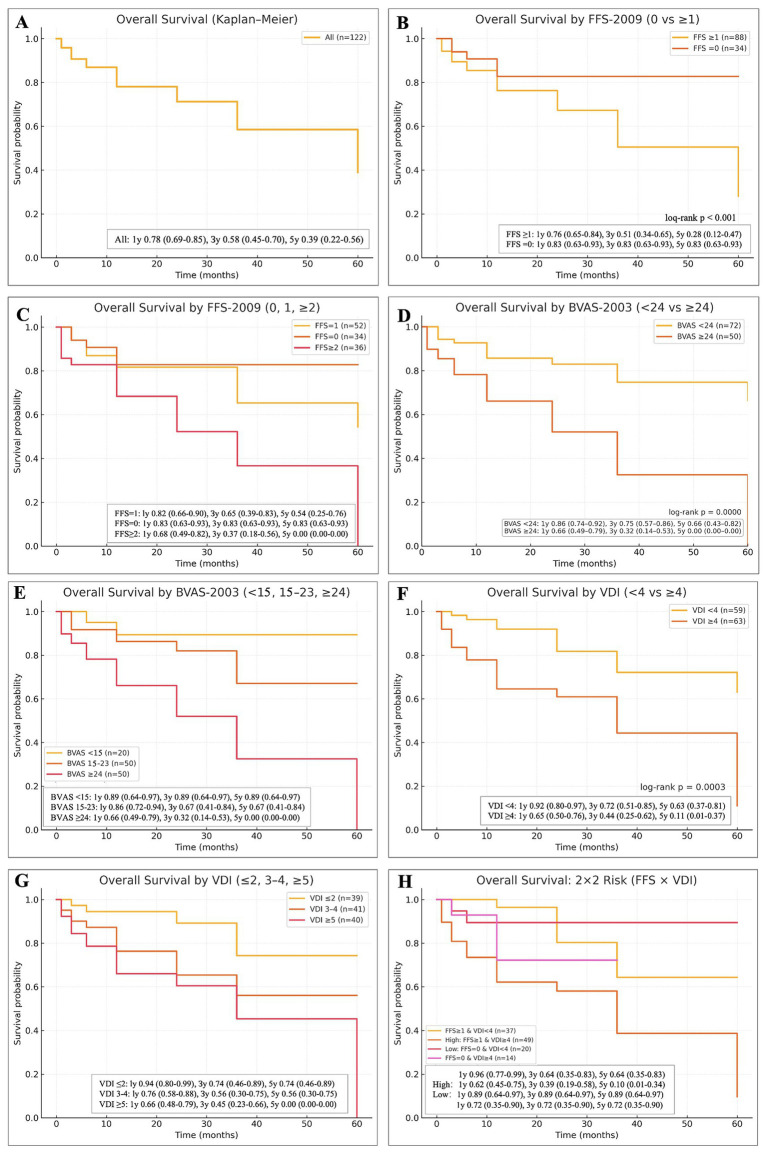
Kaplan–Meier survival curves in antineutrophil cytoplasmic antibody-associated vasculitis. **(A)** Overall survival. **(B)** Survival by Five-Factor Score 2009, 0 versus 1 or higher. **(C)** Survival by Five-Factor Score 2009, 0, 1 and 2 or higher. **(D)** Survival by Birmingham Vasculitis Activity Score 2003 below 24 versus 24 or higher. **(E)** Survival by Birmingham Vasculitis Activity Score 2003 categories. **(F)** Survival by Vasculitis Damage Index below 4 versus 4 or higher. **(G)** Survival by Vasculitis Damage Index categories. **(H)** Combined Five-Factor Score by Vasculitis Damage Index risk groups. VDI-based curves should be interpreted as accumulated damage-burden strata rather than as baseline prediction curves.

During a median follow-up of 36 months, 36 patients died. Estimated survival was 78% at 1 year, 58% at 3 years and 39% at 5 years (Figure 1A). Recorded causes of death were infection in 19 patients, active vasculitis or vasculitis-related organ failure in 11, malignancy or cardiovascular events in five and other or unspecified causes in one. Deaths were concentrated in the first 3 years after diagnosis. These survival estimates should be interpreted in the context of a severe tertiary-care cohort with frequent renal involvement and dialysis requirement. Detailed follow-up duration by final survival status was not uniformly reconstructable from the harmonized retrospective dataset and was therefore not used to support VDI-based prospective inference.

Compared with survivors, non-survivors had higher baseline BVAS-2003, higher FFS-2009 and higher accumulated VDI. They also had lower baseline hemoglobin, higher serum creatinine, more frequent hypertension and more frequent diabetes mellitus (Table 1). Disease subtype, ANCA specificity and sex were not significantly different between survivors and non-survivors. These univariable patterns indicate that mortality clustered with both vasculitis-related severity and conventional cardio-metabolic vulnerability.

### Treatment exposure, dialysis and infection-related death

Treatment data were available for most patients, although denominators varied by variable. Cyclophosphamide exposure was recorded in 53 of 111 patients and rituximab exposure in 10 of 111. Glucocorticoid pulse therapy was used in 59 of 109 patients. Differences in cyclophosphamide, rituximab, mycophenolate mofetil, pulse therapy and glucocorticoid dosing between survivors and non-survivors were not statistically significant (Table 2).

**Table 2:** Treatment exposure and renal support variables by survival status.

Characteristic	Overall (*N* = 122)	Survivors (*n* = 86)	Non-survivors (*n* = 36)	*p* value
Cyclophosphamide use	53/111 (47.7%)	39/80 (48.8%)	14/31 (45.2%)	0.833
Rituximab use	10/111 (9.0%)	9/80 (11.3%)	1/31 (3.2%)	0.278
Mycophenolate mofetil use	18/111 (16.2%)	12/80 (15.0%)	6/31 (19.4%)	0.576
Initial glucocorticoid dose, mg/day	200.0 [40.0–500.0]	200.0 [40.0–500.0]	500.0 [50.0–500.0]	0.274
Cumulative glucocorticoid dose, g	4.0 [2.7–6.6]	4.5 [3.0–7.4]	3.5 [2.5–5.8]	0.303
Glucocorticoid pulse therapy	59/109 (54.1%)	41/78 (52.6%)	18/31 (58.1%)	0.673
Dialysis	36/109 (33.0%)	20/79 (25.3%)	16/30 (53.3%)	0.011
Maintenance dialysis	18/110 (16.4%)	10/79 (12.7%)	8/31 (25.8%)	0.149

Continuous variables are presented as median [interquartile range]; categorical variables are shown as *n*/*N* (%), where *N* is the number of patients with non-missing data. *p* values compare survivors with non-survivors using available-case data.

Dialysis requirement was common and was more frequent among non-survivors than survivors. Maintenance dialysis was also more frequent among non-survivors, although this comparison did not reach statistical significance (Table 2). Infection was the leading recorded cause of death. Respiratory infection or sepsis was frequently mentioned in death summaries, but microbiological details, exact primary site beyond routine clinical description and vasculitis activity status at infection onset were not consistently documented. The available infection-related death information is summarized in Table 3.

**Table 3:** Cause-of-death summary and infection-related information available from routine records.

Recorded principal cause of death	*n* (% of deaths)	Available detail in source records	Interpretive limitation
Infection	19 (52.8)	Respiratory infection or sepsis was frequently documented in death summaries.	Detailed microbiology, exact infection site beyond routine clinical description, and active vasculitis versus remission status were not consistently available; causes were not centrally adjudicated.
Active vasculitis or vasculitis-related organ failure	11 (30.6)	Included deaths attributed clinically to active vasculitis or organ failure directly related to vasculitis.	Detailed adjudication of activity status was not performed.
Malignancy or cardiovascular events	5 (13.9)	Recorded as malignancy or cardiovascular death in clinical summaries.	Event subtype was not centrally adjudicated.
Other or unspecified	1 (2.8)	Insufficient detail for more specific classification.	Retrospective record limitation.

This table reports information that could be reliably abstracted from routine records and avoids unsupported microbiological or disease-activity classification.

### Kaplan–Meier stratification by clinical instruments

FFS-2009 stratified survival clearly. Patients with one or more FFS risk factors had worse survival than those with a score of 0 (Figure 1B). When analyzed as three categories, FFS 0, 1 and 2 or higher produced a graded survival separation, with the highest category showing the poorest prognosis (Figure 1C). These findings support the clinical relevance of baseline organ-threatening severity at diagnosis.

BVAS-2003 was associated with mortality at high values. Patients with baseline scores of 24 or higher had markedly lower survival than those with scores below 24 (Figure 1D). Tertile-based analyses showed a similar gradient, but the separation between adjacent lower categories was less pronounced (Figure 1E). Thus, very high inflammatory activity identified a high-risk subgroup, whereas BVAS alone was less robust for independent endpoint discrimination.

Accumulated damage was strongly related to survival. Patients with VDI of 4 or higher had substantially lower survival than those with lower damage scores (Figure 1F). A three-level damage classification also showed a stepwise mortality gradient (Figure 1G). Cross-classification by baseline FFS and follow-up VDI identified four clinically interpretable risk groups (Figure 1H). Because VDI was measured during follow-up rather than at a uniform early landmark, these results are best interpreted as showing that death occurred in patients with higher accumulated damage burden, not as proving that baseline damage predicted subsequent death.

### Multivariable endpoint association and discrimination

**Figure 2:**
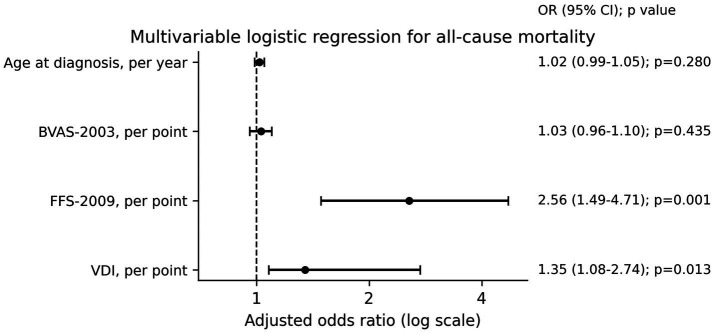
Endpoint multivariable logistic regression for all-cause mortality. Odds ratios are adjusted for age at diagnosis, Birmingham Vasculitis Activity Score 2003, Five-Factor Score 2009 and Vasculitis Damage Index. Because VDI was assessed during follow-up, the estimates should be interpreted as endpoint associations rather than time-fixed causal hazard estimates.

In the parsimonious endpoint multivariable logistic regression model, FFS-2009 and VDI retained associations with all-cause mortality. Each 1-point increase in FFS was associated with higher odds of death (adjusted odds ratio 2.56, 95% confidence interval 1.49–4.71; *p* = 0.001). Each additional VDI item was also associated with higher mortality risk (adjusted odds ratio 1.35, 95% confidence interval 1.08–2.74; *p* = 0.013). BVAS-2003 and age did not retain independent associations in this index-based endpoint model (Figure 2). Given the timing of VDI assessment, these estimates should be interpreted as endpoint associations rather than time-fixed causal hazard estimates.

**Figure 3:**
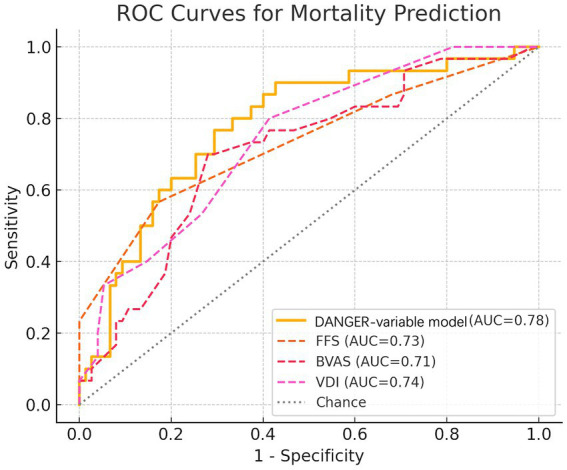
Receiver operating characteristic curves comparing discrimination for endpoint all-cause mortality. The DANGER-variable model included age, hemoglobin, serum creatinine and history of hypertension or cardiovascular disease and was not intended as validation of the original points-based DANGER score. The numerically higher AUC should be interpreted cautiously because this model contains multiple continuous predictors, whereas BVAS, FFS and VDI are individual clinical indices. AUC, area under the curve; BVAS, Birmingham Vasculitis Activity Score; FFS, Five-Factor Score; VDI, Vasculitis Damage Index.

Receiver operating characteristic analysis showed that the DANGER-variable model had the numerically highest discrimination for endpoint mortality, with an area under the curve of 0.78. VDI, FFS-2009 and BVAS-2003 had areas under the curve of 0.74, 0.73 and 0.71, respectively (Figure 3). These differences should be interpreted cautiously because the DANGER-variable model is a multivariable model using continuous baseline variables, whereas BVAS, FFS and VDI are individual clinical indices. The observed AUC pattern therefore suggests complementary information rather than definitive superiority of the DANGER framework. Sensitivity analyses excluding eosinophilic granulomatosis with polyangiitis did not materially change the direction of the main associations.

## Discussion

This two-center cohort study compared routinely available vasculitis instruments and baseline cardio-renal variables for mortality stratification in AAV. The central finding is that death clustered with baseline organ-threatening severity, accumulated damage burden and cardio-renal vulnerability more than with global activity alone. FFS-2009 at diagnosis and VDI accumulated during follow-up were associated with mortality in endpoint analyses, whereas BVAS-2003 did not retain independent discrimination after adjustment. Importantly, the study separates diagnosis-time variables from follow-up damage burden and should not be read as formal validation of a new mortality prediction model.

The main novelty is a construct-sensitive framework for interpreting commonly used AAV measures. In clinical practice, BVAS, FFS and VDI are sometimes discussed as if they were interchangeable severity scores. They are not. BVAS quantifies current inflammatory activity, FFS summarizes baseline prognostic severity and VDI records irreversible damage. By comparing these instruments within the same cohort and adding DANGER-related variables, our study highlights that mortality stratification requires both disease-centered assessment and host-vulnerability assessment.

The association between VDI and death is clinically plausible because irreversible organ damage and treatment-related harm contribute to poor outcomes in AAV (10, 11, 24, 25). However, VDI timing is the most important interpretive issue in this study. VDI was updated at the last available follow-up or immediately before death; it therefore cannot be considered a pure baseline predictor. Patients who survive longer have more opportunity to accrue damage, and patients who die may have damage recorded close to the outcome. We therefore interpret VDI as accumulated damage burden during the observed disease course. The finding remains clinically useful because high damage burden identifies a population requiring closer monitoring, infection prevention, renal and cardiovascular co-management and steroid-sparing strategies, but it requires prospective validation using standardized 6- and 12-month damage assessments.

BVAS-2003 remains essential for defining active disease and guiding treatment intensity, but our data suggest that its role as an independent endpoint mortality discriminator is more limited. Very high baseline activity identified a poor-prognosis group, yet its independent effect attenuated after accounting for FFS and damage. This pattern is consistent with the broader view that not all active manifestations carry equal prognostic weight; organ-threatening features and accumulated damage may be more relevant for survival than the global count of active items (13, 26). At the same time, other cohorts have reported clinically meaningful relationships between activity scores, conventional baseline indices and early mortality, underscoring that our findings should be interpreted within the context of a severe tertiary-care cohort rather than as a universal hierarchy of prognostic instruments (13, 14).

The DANGER-variable model performed well in this cohort, but its interpretation should be balanced. We included these variables because a recently proposed DANGER score in ANCA-associated renal vasculitis emphasized age, hemoglobin, renal function and cardiovascular history as compact markers of mortality risk (18). In the present study, we did not apply or recalibrate the original points-based DANGER score. Instead, we used its component variables in a logistic model to test whether a simple baseline cardio-renal vulnerability construct could complement vasculitis-specific instruments. The AUC was numerically higher than those of individual indices, but the comparison is not equivalent because the DANGER-variable model contains multiple continuous predictors, while BVAS, FFS and VDI were evaluated as individual scores. Therefore, the result supports complementarity rather than clear superiority.

The observed survival was lower than that reported in some contemporary cohorts and long-term AAV studies (2, 3). Several factors may explain this pattern. First, renal involvement and dialysis requirement were common. Second, deaths clustered early, and infection was the leading recorded cause of death. Infection remains a major early hazard in AAV, particularly in patients exposed to high-dose glucocorticoids and intensive immunosuppression (1, 6, 7). Third, the cohort came from tertiary centers and may have included a higher proportion of severe or referred cases. Fourth, treatment exposure data did not indicate systematic undertreatment of non-survivors, supporting the interpretation that confounding by indication and baseline disease severity likely contributed to the high mortality. These considerations are important when comparing survival estimates across population-based, nephrology-based and tertiary rheumatology cohorts.

The practical implication is a two-domain risk framework. FFS should anchor baseline prognostic assessment at diagnosis, whereas VDI should be used longitudinally to monitor accumulated harm. A high FFS should prompt early risk-adapted supportive care, including infection prevention, renal protection, cardiovascular assessment and careful glucocorticoid exposure management, consistent with current treatment recommendations for AAV (4, 5). A rising or high VDI during follow-up should trigger reassessment of treatment toxicity, irreversible organ damage and the balance between disease control and harm reduction.

This study has limitations. The design was retrospective, and treatment exposure was incompletely captured for some variables. Causes of death were not centrally adjudicated. The number of deaths limited model complexity and precluded a fully adjusted model including all conventional risk factors. The index-based logistic model should therefore be interpreted as an exploratory endpoint association model, not as a definitive causal prediction model. A time-to-event Cox model would generally be desirable for baseline mortality predictors, but a standard Cox model including VDI as if it were a baseline covariate would be inappropriate, and serial VDI dates were unavailable for time-dependent Cox modeling. VDI was unavailable at standardized 6- or 12-month landmarks, creating potential survivorship bias, reverse causation and endpoint-proximity bias. Follow-up duration by final survival status was not uniformly reconstructable from the harmonized retrospective dataset, which may further affect damage accrual and VDI-related ROC comparisons. Infection-related deaths were frequent, but detailed site, microbiology and active-versus-remission status were not consistently documented. Some cut-offs were cohort-informed and require external validation. Finally, the DANGER-variable model evaluated discrimination of selected component variables in this cohort but did not validate the original points-based DANGER score.

In conclusion, mortality stratification in AAV should distinguish current inflammatory activity, baseline organ-threatening severity, longitudinal accumulated damage and baseline cardio-renal vulnerability. In this severe two-center cohort, FFS-2009 and final VDI were more informative for endpoint mortality than BVAS-2003 alone, while DANGER-related cardio-renal variables showed complementary discrimination. Prospective multicenter cohorts with standardized early VDI assessment, group-specific follow-up reporting, adjudicated causes of death and formal validation of baseline risk models are needed to determine whether risk-adapted monitoring can reduce infection-related and renal-related mortality.

## Data Availability

The raw data supporting the conclusions of this article will be made available by the authors, without undue reservation.
